# Health Inequities in the USA: a Role for Dietary Acid Load? Results from the National Health and Nutrition Examination Surveys

**DOI:** 10.1007/s40615-022-01462-9

**Published:** 2022-11-23

**Authors:** Maximilian Andreas Storz

**Affiliations:** Department of Internal Medicine II, Centre for Complementary Medicine, Faculty of Medicine, Freiburg University Hospital, University of Freiburg, Freiburg, Germany

**Keywords:** Health inequities, Racial and ethnic health disparities, Dietary acid load, Potential renal acid load, Net endogenous acid production

## Abstract

**Background:**

Cardiovascular disease and obesity affect racial and ethnic minorities disproportionally. Public health research suggests that suboptimal diet is an important contributor to health disparities. Limited evidence points at an increased dietary acid load (DAL) in certain ethnic groups. DAL is determined by the balance of acidifying foods and alkaline foods, and elevated DAL scores have been associated with numerous chronic lifestyle-related conditions. The present analysis investigated DAL scores among ethnic groups in the USA.

**Methods:**

Using cross-sectional data from the National Health and Nutrition Examination surveys (NHANES, 2007–2016), we contrasted several markers of DAL (potential renal acid load (PRAL) and net endogenous acid production (NEAP)) between Non-Hispanic Whites, Non-Hispanic Blacks, Mexican Americans, Other Hispanics, and Other Race. The comparison included crude scores and adjusted scores following multivariate linear regression.

**Results:**

The sample for this analysis comprised 19,565 participants, which may be extrapolated to represent 156,116,471 United States Americans. When compared to Non-Hispanic Whites, Non-Hispanic Blacks and Mexican Americans had significantly higher crude DAL scores. PRAL_R_ was highest in Mexican Americans (20.42 (0.61) mEq/day), followed by Non-Hispanic Blacks (17.47 (0.42) mEq/day). Crude NEAP_F_ was highest in Non-Hispanic Blacks (64.66 (0.43) mEq/day), and almost 9 mEq/day higher compared to Non-Hispanic Whites (55.78 (0.39) mEq/day). Multivariate linear regression adjusting for confounders revealed comparable interracial DAL differences.

**Conclusions:**

We found significant DAL differences across the investigated ethnic groups. Whether these differences potentially play a role in population health inequity in the USA will be subject to additional research.

**Graphical Abstract:**

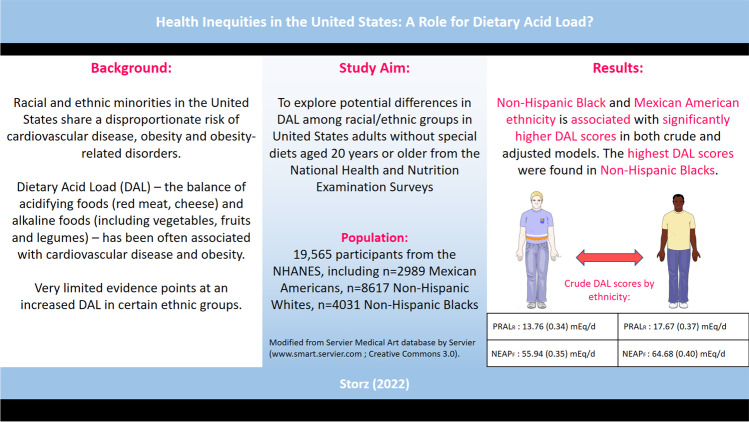

**Supplementary Information:**

The online version contains supplementary material available at 10.1007/s40615-022-01462-9.

## Background

Health equity is a frequently cited goal of public health in the USA. In recent years, public health leaders have placed greater emphasis on achieving equity in population health [1–3], and implemented numerous approaches to combat the medical and social burden resulting from racial and ethnic disparities [4]. Nevertheless, a recent study by Zimmermann and Anderson suggested a clear lack of progress on health equity during the past 25 years [3].

For racial and ethnic minorities in the USA, health disparities take on many forms, including (but not limited to) higher premature death rates and a higher incidence of chronic diseases [5]. Cardiovascular disease (CVD), obesity, and obesity-related conditions affect racial and ethnic minorities disproportionally [6–8].

Non-Hispanic Blacks, for example, have a substantially higher CVD mortality compared with other races/ethnic groups [9]. The age-adjusted mortality rate from CVD of Black men in 2018 was 260 per 100,000, compared to 206 per 100,000 in Non-Hispanic White men. Moreover, all-cause mortality rates among Black populations was 24% higher than among White populations in the 30 most populous United States cities [10]. Analyses of maternal mortality disparities revealed that the maternal mortality rate for Non-Hispanic Black women was 3.55 times that for non-Hispanic White women [11].

Minority groups also share a disproportionate risk of obesity and obesity-related diseases. Odds of obesity were shown to be significantly higher in Non-Hispanic Blacks (OR = 1.97) and Hispanics (OR = 1.81) compared to Non-Hispanic Whites [12].

Socioeconomic factors, fewer health-promoting resources, differences in social norms, lifestyle-related aspects, genetics and psychological factors have been discussed as determinants of obesity and CVD disparities [13, 14, 16]. Public health research shows that suboptimal diet is another important contributor to health disparities among racial and ethnic minorities [17]. Non-Hispanic Blacks and Hispanics tend to have a greater prevalence of poor diet scores than Non-Hispanic Whites [18]. Analyses by Brown et al. suggested that in US adults without prevalent cardiovascular disease, the number of Non-Hispanic Blacks with a poor diet was greater than the number of Non-Hispanic Whites by a magnitude of 6.8 to 11.7% percentage points from 1988 to 2010 [6].

Very limited evidence points at an increased dietary acid load (DAL) in certain ethnic groups [19]. DAL is determined by the balance of acidifying foods (including red meat and cheese) and alkaline foods (such as vegetables, fruits, and legumes) [20]. Diet can markedly affect acid–base status [21], and an altered acid–base homeostasis in the form of an acidifying diet has been shown to modulate molecular activity, including adrenal glucocorticoid secretion, adipocyte cytokine signaling, and altered insulin sensitivity [22]. An alkaline diet, to the contrary, has been associated with a reduction of morbidity and mortality from chronic diseases [23].

Contemporary Western diets typically produce a total acid load ranging from 50 to 80 mEq per day [24, 25]. Crews et al. suggested that DAL is substantially higher in Non-Hispanic Blacks; however, their analysis was limited to patients with chronic kidney disease [19]. Whether there are also DAL differences among other racial and ethnic groups in the general population in the USA has not yet been analyzed in large population-based studies. A high DAL induces a low-grade metabolic acidosis state, which has been associated with the development of metabolic alterations such as insulin resistance, diabetes, and cardiovascular disease; conditions that are more frequently found in ethnic minorities in the USA [26]. In light of the numerous chronic conditions associated with pathologically elevated DAL scores [27–30], DAL is an important modifiable lifestyle risk factor.

In this context, we used data from the National Health and Nutrition Examination Surveys (NHANES) to investigate two main questions: (1) are there differences in DAL among racial/ethnic groups in the USA, and (2) which groups are disproportionally affected? In this study, we hypothesized that compared to Non-Hispanic Whites, other ethnic groups in the USA yield higher markers of DAL.

## Methods

### Study Population and Design

The present analysis is based on aggregated population-based cross-sectional data from the NHANES [31]. The NHANES is an ongoing cross-sectional survey in the United States of America that uses a complex, stratified, multistage probability sampling design [32]. It was designed to assess the health and nutritional status of non-institutionalized adults in the USA and is one of several large health‐related programs conducted by the National Center for Health Statistics in the USA.

NHANES study and survey protocols were approved by the Research Ethics Review Board of the National Center for Health Statistics, and written informed consent was obtained from all participants [33]. NHANES data is publicly available but without personal identifiable information. We carried out all methods in full accordance with relevant guidelines and regulations.

For this study, we included all participants aged 20 years or older who participated in the continuous NHANES survey cycles of 2007–2008 through 2015–2016 to increase the potential sample size for analyses stratified by population subgroups. We constructed appropriate sample weights for the combined NHANES cycles based on National Center for Health Statistics guidelines [34].

For the present analysis, we merged multiple NHANES modules of interest. Participant characteristics were obtained from the demographics module and included age, sex, race/ethnicity, marital status, annual household income, and education level. Nutrient intake data was obtained from the dietary interview component. This module used a computerized 24-h dietary recall method to estimate energy and nutrient intake for all participants. The examination protocol and the data collection methods may be obtained from the NHANES dietary interviewer’s procedure manual [35]. Anthropometric data was obtained from the body measures module and included height, weight, and body mass index (BMI).

### Dietary Acid Load Markers

In the NHANES, nutritional assessment was conducted through two repeated 24-h dietary recalls [36]. This is a retrospective and quantitative method to gather information about foods and beverages consumed by the participants in the 24 h prior to their visit. For this analysis, we relied solely on dietary data from day 1, and calculated DAL based on nutrient intake data.

The methods for estimating DAL have been described in detail elsewhere [37–39]. In brief, we used the formulas by Remer et al. and Frassetto et al. to estimate DAL scores from nutrient intake [40, 41]. All formulas are commonly used in epidemiological and clinical research. Their performance and accuracy have been discussed in detail by Parmenter et al. [42]. Remer and Manz estimated potential renal acid load (PRAL_R_) based on intestinal absorption rates of potassium, phosphate, magnesium, calcium, and protein [43]. Their formula is as follows:

$$\begin{array}{c}{\mathrm{PRAL}}_{\mathrm R}\;\left(\mathrm{mEq}/\mathrm{day}\right)=\left(0.49\times\mathrm{total}\;\mathrm{protein}\;\left(\mathrm g/\mathrm{day}\right)\right)+\left(0.037\times\mathrm{phosphorus}\;\left(\mathrm{mg}/\mathrm{day}\right)\right)-\left(0.021\times\right.\\\mathrm{potassium}\;\left(\left.\mathrm{mg}/\mathrm{day}\right)\right)-\left(0.026\times\mathrm{magnesiun}\;\left(\mathrm{mg}/\mathrm{day}\right)\right)-\left(0.013\times\mathrm{calcium}\;\left(\mathrm{mg}/\mathrm{day}\right)\right)\end{array}$$

This formula has been cross-validated and considers ionic dissociation and sulfur metabolism. Frassetto et al. estimated net endogenous acid production (NEAP) based on protein and potassium intake:

$${\mathrm{NEAP}}_{\mathrm F}\;\left(\mathrm{mEq}/\mathrm{day}\right)=\left(54.4\times\mathrm{protein}\;\left(\mathrm g/\mathrm{day}\right)/\mathrm{potassium}\;\left(\mathrm{mEq}/\mathrm{day}\right)\right)-10.2$$

The third employed formula (NEAP_R_) also stems from the work of Remer et al. and uses anthropometric measures and nutrient quantities of cations and anions derived from dietary intake. NEAP_R_ was estimated as follows:

$$\mathrm{Estimated}\;{\mathrm{NEAP}}_{\mathrm R}\left(\mathrm{mEq}/\mathrm{day}\right)=\mathrm{PRAL}\;\left(\mathrm{mEq}/\mathrm{day}\right)+\mathrm{OAes}t\;\left(\mathrm{mEq}/\mathrm{day}\right)$$

OAest (mEq/day) was calculated as follows:

$$\mathrm{Individual}\;\mathrm{body}\;\mathrm{surface}\;\mathrm{area}\;\times41/1.73$$

To estimate individual body surface area, we used the Du Bois and Du Bois formula [44]. Each of the aforementioned formulas has its strengths and limitations; thus, we decided to use them in this particular combination to allow for a more detailed DAL quantification [42].

### Inclusion and Exclusion Criteria

Inclusion criteria were as follows: age ≥ 20 years, available demographic data, plausible energy intake data (based on Willett’s criteria) [45], available anthropometric data, and plausible DAL scores. Participants that indicated consumption of a special diet (e.g. a low-carbohydrate diet, gluten-free diet) were excluded to reduce the likelihood of selection bias. Special diet status was assessed based on the NHANES “DRQSDIET” variable, inquiring about special diets for weight loss or other health reasons [37].

### Statistical Analysis

We used the STATA 14 statistical software (StataCorp. 2015. Stata Statistical Software: Release 14. College Station, TX: StataCorp LP) for our statistical analysis.

Histograms, box plots, and subpopulation summary statistics were used to check for normality of the data. Normally distributed variables were described with their mean and standard error in parenthesis, whereas categorical variables were described with their weighted proportions and standard error in parenthesis. We estimated standard errors using Taylor series linearization to account for the complex NHANES sampling design. Appropriate sample weights were used to account for differential nonresponse and/or non-coverage, to adjust for planned oversampling of some groups, and to generate weighted percentages and means that are representative of the noninstitutionalized civilian population. Since we appended five different NHANES cycles (2007–2008 through 2015–2016), we generated a 10‐year weight for dietary data (wtdrd10yr = wtdrd1/5).

Based on the most recent NCHS data presentation standards for proportions [46], we checked all weighted proportions for potential unreliability using the user-written post-estimation command “kg_nchs” in Stata [47]. Unreliable proportions were flagged with superscript letters.

Stata’s Rao–Scott test (a design-adjusted version of the Pearson chi-square test) and multivariate linear regression analyses (followed by adjusted Wald tests and Stata’s margins function) were used to test for potential differences between races/ethnicities. All multivariate linear regression models were constructed based on the recommendations of West, Berglund, and Heeringa [48].

In a first step, we conducted exploratory bivariate analyses to investigate potential candidate predictors with a significant relationship with DAL scores (PRAL_R_ and NEAP_F_). We included only predictor variables of scientific relevance and with a bivariate relationship of significance < 0.25 with the response variables in the initial model. Using *t*-tests for individual coefficients and Wald tests for multiple coefficients, we verified the importance of the included predictor variables and assessed potential changes in all predictor variables in the multivariate model. Finally, we tried to improve the overall fit of the model using established survey data analyses techniques [48]. Post regression, marginsplots were used to graph statistics from fitted models. We used a *p*-value < 0.05 as a cutoff for statistical significance.

## Results

The total sample for this analysis comprised 19,565 participants, which may be extrapolated to represent 156,116,471 United States Americans. The unweighted numbers of participants were as follows: *n* = 2989 Mexican Americans, *n* = 1951 Other Hispanics, *n* = 8617 Non-Hispanic Whites, *n* = 4031 Non-Hispanic Blacks, and *n* = 1977 Other Race (including multi-racial).

Table 1 shows sample characteristics by race/ethnicity. Mean age of Non-Hispanic Whites was significantly higher than for the other groups 49.01 (0.32). Significant intergroup differences were also found with regard to the weighted proportions of males and females (Table 1). The weighted proportion of obese participants was significantly higher in Mexican Americans (43.38% (1.19)) and Non-Hispanic Blacks (44.58% (1.27)). Significant intergroup differences in the weighted proportions were also found regarding marital status, educational level, and annual household income.

**Table 1:** Sample characteristics by race/ethnicity

	Mexican American	Other Hispanic	Non-Hispanic White	Non-Hispanic Black	Other Race ^a^	*p*-value
Sex						
Male Female	52.94% (0.93) 47.06% (0.79)	48.36% (1.20) 51.64% (1.20)	50.12% (0.58) 49.88% (0.58)	45.38% (0.88) 54.62% (0.88)	49.03% (1.27)^c^ 50.97% (1.27)^c^	*p* < 0.001^b^
Age (years)	39.61 (0.45)	41.36 (0.56)	49.01 (0.32)	43.88 (0.49)	43.83 (0.58)	*p* < 0.001^c^
Marital status						
Married/living with partner Widowed/divorced/separated Never married	69.31% (1.53) 12.99% (0.80) 17.70% (1.29)	58.85% (1.71) 17.28% (1.20) 23.87% (1.23)	65.18% (0.92) 18.73% (0.58) 16.09% (0.85)	41.82% (1.27) 22.64% (0.96) 35.54% (1.26)	64.60% (1.75)^c^ 12.74% (1.36)^c^ 22.67% (1.46)^c^	*p* < 0.001^b^
Annual household income						
< 20,000 US$ > 20,000 US$	23.10% (1.55) 76.90% (1.55)	22.81% (1.53) 77.19% (1.53)	11.66% (0.78) 88.34% (0.78)	26.09% (1.30) 73.91% (1.30)	13.16% (1.04) 86.84% (1.04)	*p* < 0.001^b^
Education level						
Less than 9th grade 9–11th grade High school graduate/GED^d^ Some college or AA degree College graduate or above	24.84% (1.30) 21.75% (1.01) 20.23% (0.98) 24.02% (1.10) 9.16% (0.72)	14.16% (1.36) 15.88% (1.20) 23.19% (1.27) 29.55% (1.82) 17.21% (1.57)	02.25% (0.28) 08.46% (0.72) 22.93% (0.88) 32.59% (0.76) 33.77% (1.37)	3.48% (0.38) 17.56% (0.89) 27.38% (0.96) 34.70% (0.87) 16.88% (1.09)	4.19% (0.55)^c^ 7.34% (0.91)^c^ 16.18% (1.30)^c^ 27.65% (1.68)^c^ 44.65% (2.11)^c^	*p* < 0.001^b^
BMI						
< 18.50 ≥ 18.50 and < 25.00 ≥ 25.00 and < 30.00 ≥ 30	0.62% (0.20) 20.07% (1.26) 35.93% (1.18) 43.38% (1.19)	0.80% (0.31) 27.06% (1.27) 35.86% (1.46) 36.28% (1.82)	1.64% (0.19) 31.78% (0.74) 34.29% (0.73) 32.28% (0.71)	2.00% (0.29) 24.65% (0.82) 28.77% (0.96) 44.58% (1.27)	3.14% (0.58)^c^ 47.60% (1.45)^c^ 29.11% (1.51)^c^ 20.15% (1.78)^c^	*p* < 0.001^b^

Weighted proportions. Total number of unweighted observations: 19,565. Continuous variables shown as mean (standard error). Categorical variables shown as weighted proportion (standard error). All weighted proportions can be considered reliable, as peer recent NCHS Guidelines. ^a^Includes multi-racial; ^b^based on Stata’s design-adjusted Rao–Scott test, ^c^based on regression analyses followed by adjusted Wald tests, ^d^or equivalent

Table 2 displays nutrient and total energy intake by race/ethnicity. Significant intergroup differences were found across all 5 groups. Mexican Americans had the highest total energy intake (2262.30 (21.98) kcal/day) and the highest total protein intake (89.22 (1.03) g/day). The lowest protein intake was found in Non-Hispanic Blacks (78.66 (0.66)). The latter also had a significantly lower total potassium intake (2351.63 (21.55) mg/day) compared to the other groups. Moreover, Non-Hispanic Blacks also had the lowest magnesium (264.54 (2.93) mg/day) and phosphorus intake (1233.90 (9.58) mg/day). Sodium intake exceeded 3000 mg/day in all five groups (Table 2).

**Table 2:** Nutrient intake and energy intake by race/ethnicity

	Mexican American	Other Hispanic	Non-Hispanic White	Non-Hispanic Black	Other Race	*p*-value
Energy intake (kcal/day)	2262.30 (21.98)	2106.14 (20.50)	2185.97 (10.39)	2152.98 (17.19)	2014.39 (27.93)	*p* < 0.001^a^
Protein intake (g/day)	89.22 (1.03)	83.74 (1.17)	82.19 (0.58)	78.66 (0.66)	80.42 (0.98)	*p* < 0.001^a^
Carbohydrate intake (g/day)	278.79 (3.16)	264.48 (2.61)	259.82 (1.42)	260.93 (2.32)	254.40 (3.72)	*p* < 0.001^a^
Fat intake (g/day)	84.13 (1.11)	75.70 (1.18)	84.70 (0.54)	82.68 (0.88)	72.09 (1.38)	*p* < 0.001^a^
Potassium intake (mg/day)	2737.99 (35.90)	2610.72 (38.31)	2768.40 (20.13)	2351.63 (21.55)	2647.51 (34.28)	*p* < 0.001^a^
Sodium intake (mg/day)	3657.78 (44.55)	3390.79 (45.23)	3581.21 (22.53)	3415.82 (29.04)	3773.50 (66.55)	*p* < 0.001^a^
Magnesium intake (mg/day)	318.35 (4.29)	294.06 (4.35)	308.65 (2.67)	264.54 (2.93)	309.73 (4.67)	*p* < 0.001^a^
Phosphorus intake (mg/day)	1503.79 (16.33)	1342.24 (18.11)	1414.56 (8.83)	1233.90 (9.58)	1281.08 (16.65)	*p* < 0.001^a^
Calcium intake (mg/day)	1012.73 (14.10)	928.46 (15.21)	1006.84 (8.42)	805.09 (11.09)	821.58 (15.20)	*p* < 0.001^a^

Unweighted number of observations: 19,565. Continuous variables shown as mean (standard error). ^a^Based on regression analyses followed by adjusted Wald tests

Table 3 shows crude dietary acid load scores by race/ethnicity. Mexican Americans had the highest mean PRAL_R_ scores (20.42 (0.61) mEq/day), followed by Non-Hispanic Blacks (17.47 (0.42) mEq/day). A comparable sequence was found with regard to NEAP_R_. Mexican Americans had the highest mean NEAP_R_ scores (64.64 (0.67) mEq/day), followed by Non-Hispanic Blacks (63.82 (0.47) mEq/day). For NEAP_F_, the highest average scores were found in Non-Hispanic Blacks (64.66 (0.43) mEq/day), followed by Mexican Americans (61.70 (0.69) mEq/day). The mean PRAL_R_ score difference between Mexican Americans and Non-Hispanic Whites was approximately 7.06 mEq/day. When compared to Non-Hispanic Whites, mean unadjusted NEAP_F_ scores were almost 9 mEq/day higher in Non-Hispanic Blacks.

**Table 3:** Crude dietary acid load scores in mEq/day by race/ethnicity

	Mexican American	Other Hispanic	Non-Hispanic White	Non-Hispanic Black	Other Race	*p*-value
PRAL_R_	20.42 (0.61)	16.15 (0.69)	13.36 (0.37)	17.47 (0.42)	12.48 (0.62)	*p* < 0.001 ^a^
NEAP_R_	64.64 (0.67)	59.99 (0.79)	59.00 (0.41)	63.82 (0.47)	54.82 (0.69)	*p* < 0.001 ^a^
NEAP_F_	61.70 (0.69)	61.44 (0.76)	55.78 (0.39)	64.66 (0.43)	57.59 (0.68)	*p* < 0.001 ^a^

Unweighted number of observations: 19,565. Continuous variables shown as mean (standard error). ^a^Based on regression analyses followed by adjusted Wald tests

Tables 4 and 5 show the final multivariate linear regression models examining the associations between race/ethnicity and dietary acid load (as assessed by PRAL_R_ (Table 4) and NEAP_F_ (Table 5)). In model 1, we adjusted for age and sex. In model 2, we additionally adjusted for BMI and total energy intake. As shown in Table 4, Mexican Americans and Non-Hispanic Blacks yielded significantly higher PRAL_R_ values after adjustment for confounders in both regression models. Mexican American ethnicity increased PRAL_R_ values by more than 4.37 mEq/day (*p* < 0.001) in model 1. After adjustment for BMI and total energy intake, this association was still significant (3.91 mEq/day, *p* < 0.001). Non-Hispanic Black ethnicity increased PRAL_R_ values by more than 3.16 mEq/day in both models.

**Table 4:** Multivariate linear regression models examining potential associations between race/ethnicity and potential renal acid load (as assessed by PRAL_R_)

Independent variables	*β*	linearized SE	*p*	*β*		linearized SE	*p*
	Model 1				Model 2		
Sex							
Female	− 8.46	0.45	< 0.001	− 3.14		0.46	< 0.001
Age							
30–39 years 40–49 years 50–59 years 60–69 years 70 years or older	− 1.38 − 2.97 − 7.67 − 10.00 − 12.62	0.82 0.69 0.84 0.75 0.71	0.085 < 0.001 < 0.001 < 0.001 < 0.001	− 2.16 − 3.43 − 7.13 − 8.32 − 9.14		0.75 0.69 0.82 0.67 0.61	0.005 < 0.001 < 0.001 < 0.001 < 0.001
Ethnicity							
Mexican American Other Hispanic Non-Hispanic Black Other Race ^a^	4.37 0.94 3.24 − 2.14	0.74 0.75 0.60 0.68	< 0.001 0.212 < 0.001 0.002	3.91 1.84 3.16 0.37		0.68 0.74 0.56 0.73	< 0.001 0.015 < 0.001 0.618
Body mass index							
Underweight (BMI ≤ 18.49) Overweight (BMI 25–29) Obese (BMI ≤ 30)				− 1.23 2.03 4.64		1.33 0.54 0.57	0.358 < 0.001 < 0.001
Energy intake (kcal)				0.009		0.0003	< 0.001

^a^Includes multi-racial. Significant regression equations were found for both models: *F*(10,70) = 79.26 (model 1) and *F*(14,66) = 107.44 (model 2), respectively, with a *p*-value < 0.001 for both, and with *R*^2^ values of 0.086 and 0.018, respectively. Reference categories were as follows: age 20–29 years, Non-Hispanic White, BMI ≥ 18.50 and < 25.00, energy intake

**Table 5:** Multivariate linear regression models examining potential associations between race/ethnicity and net endogenous acid production (as assessed by NEAP_F_)

Independent variables	*β*	linearized SE	*p*	*β*		linearized SE	*p*
	Model 1				Model 2		
Sex							
Female	− 5.05	0.46	< 0.001	− 4.20		0.50	< 0.001
Age							
30–39 years 40–49 years 50–59 years 60–69 years 70 years or older	− 3.62 − 5.81 − 9.22 − 11.62 − 14.01	0.84 0.75 0.88 0.78 0.76	< 0.001 < 0.001 < 0.001 < 0.001 < 0.001	− 4.25 − 6.44 − 9.80 − 12.17 − 13.99		0.82 0.76 0.88 0.75 0.73	< 0.001 < 0.001 < 0.001 < 0.001 < 0.001
Ethnicity							
Mexican American Other Hispanic Non-Hispanic Black Other Race^a^	3.26 3.68 7.78 0.45	0.83 0.82 0.62 0.72	< 0.001 < 0.001 < 0.001 0.530	2.59 3.53 7.25 1.37		0.82 0.83 0.60 0.76	0.002 < 0.001 < 0.001 0.075
Body mass index							
Underweight (BMI ≤ 18.49) Overweight (BMI 25–29) Obese (BMI ≤ 30)				− 0.79 1.61 5.04		1.40 0.63 0.64	0.573 0.013 < 0.001
Energy intake (kcal)				0.0013		0.0003	< 0.001

^a^Includes multi-racial. Significant regression equations were found: *F*(10,70) = 80.12 (model 1) and *F*(14,66) = 68.29 (model 2), respectively, with a *p*-value < 0.001 for both, and with *R*^2^ values of 0.069 and 0.079, respectively

We found significantly higher NEAP_F_ values in Non-Hispanic Blacks (+ 7.78 in model 1 and 7.25 mEq/day in model 2, respectively) compared to the reference group. Energy intake and obesity were also associated with high DAL scores (Model 2, Tables 4 and 5). Our initial hypothesis was thus partially supported with regard to Mexican Americans and Non-Hispanic Blacks.

**Fig. 1:**
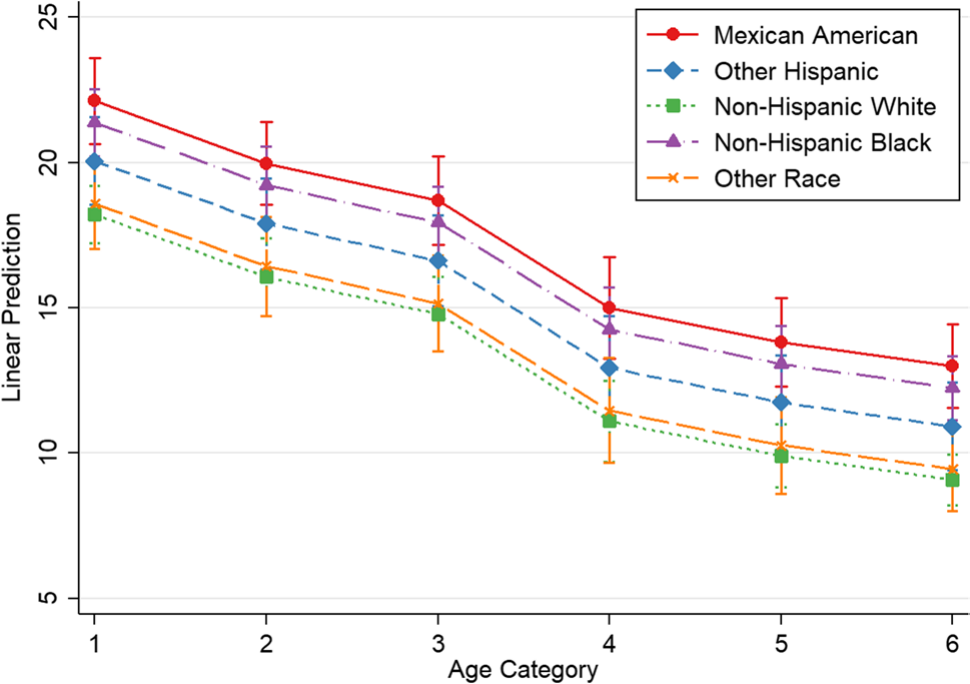
Marginsplot—potential renal acid load by race/ethnicity. Plot of marginal predicted values based on regression model 2, illustrating differences in the relationship of PRAL_R_ and age, depending on race/ethnicity

**Fig. 2:**
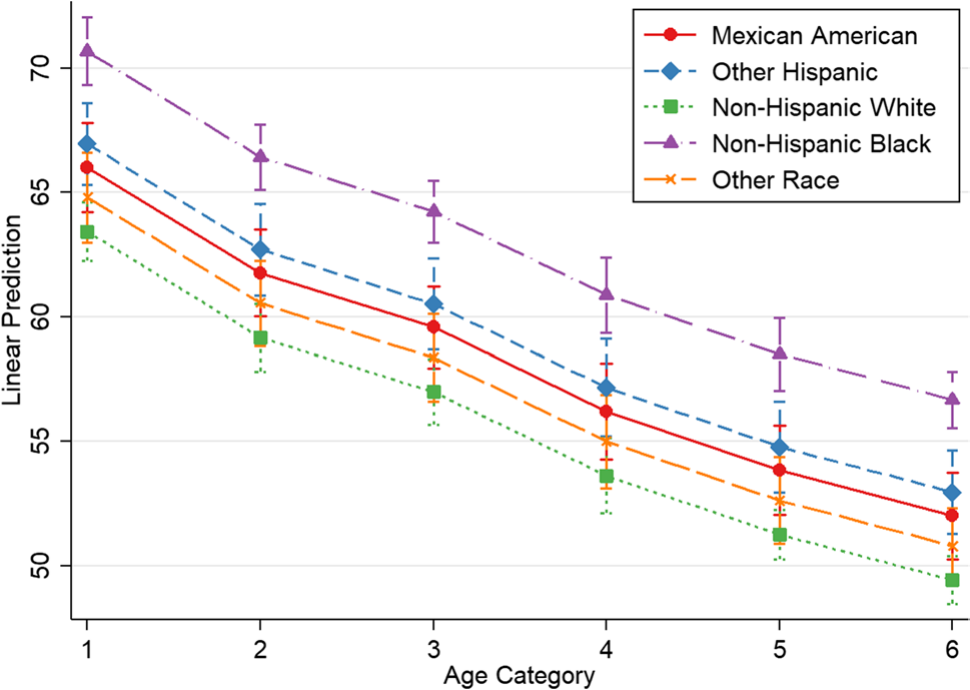
Marginsplot—net endogenous acid production by race/ethnicity. Plot of marginal predicted values based on regression model 2, illustrating differences in the relationship of NEAP_F_ and age, depending on race/ethnicity

Marginsplots were used to graph statistics from the fitted models. Marginal predicted values of PRAL_R_ are shown in Fig. 1 by race/ethnicity for each age category (based on model 2, Table 4). In a similar manner, Fig. 2 shows marginal predicted values of NEAP_F_ by race/ethnicity for each age category (based on model 2, Table 5).

We refrained from constructing the same models for formula NEAP_R_, as the estimation formula is based on anthropometric data. Using model 2 for NEAP_R_ may thus have introduced multicollinearity problems and therefore we decided to adjust only for age, sex, and ethnicity. Results may be obtained from the supplementary Table 1.

## Discussion

Using data from the NHANES, the present study sought to analyze potential differences in DAL scores among racial/ethnic groups in the USA. When compared to Non-Hispanic Whites, Non-Hispanic Blacks and Mexican Americans had significantly higher (crude) DAL scores. PRAL_R_ was highest in Mexican Americans (20.42 (0.61) mEq/day), followed by Non-Hispanic Blacks (17.47 (0.42) mEq/day). Crude NEAP_F_ was highest in Non-Hispanic Blacks (64.66 (0.43) mEq/day), and almost 9 mEq/day higher compared to Non-Hispanic Whites.

A high DAL has been associated with an elevated cardio-metabolic risk and may unfavorably affect lipid metabolism [49]. Han et al. and Murakami et al. both reported positive associations between higher PRAL-values and the concentrations of total cholesterol and low-density lipoprotein [30, 50]. Higher DAL scores were also associated with higher HbA1c concentrations and elevated triacylglycerol (TAG) concentrations [51]. Moreover, a high acid load burden from diet was associated with a higher obesity prevalence, and has thus been discussed as a potential risk factor for the development of metabolic disorders [52, 53].

The elevated DAL scores in Non-Hispanic Blacks and Mexican Americans reported in our study warrant further discussion—particularly in the potential context of chronic diseases. In 2018, African Americans were 30% more likely to die from heart disease than non-Hispanic Whites [54]. Mexican Americans on the other hand were significantly more likely to suffer from obesity or diabetes than non-Hispanic Whites [55, 56].

The numerous reasons that may contribute to these ethnic/racial disparities include socioeconomic factors, a lack of health-promoting resources in certain areas and among certain minority groups, lifestyle-related factors, genetics, and varying social norms [13, 14, 16]. Whether a high DAL may be one of the many contributors in this multifactorial process has not yet been scientifically discussed. Notably, the present analysis *does not allow* for any causal attributions but suggests that DAL differences across ethnic/racial groups exist.

The low-grade metabolic acidosis state induced by a high DAL has been associated with the development of metabolic alterations such as insulin resistance, diabetes, and cardiovascular disease [26]. Said conditions are more frequently found in ethnic minorities in the USA. It is not inconceivable that DAL differences could potentially play a role in ethnic/racial disparities in many chronic conditions, and our analysis builds the foundation for future research in this area.

Disparities in chronic diseases between various segments of the population, such as racial and ethnic groups, have increasingly become a major focus of public health research [57,58]. Individuals with a greater number of chronic conditions often present with poorer overall dietary intakes [59]. Non-Hispanic Whites were shown to have a higher diet quality (as assessed by the Alternative Healthy Eating Index 2010) compared to Hispanics and African Americans [60]. Recent trends in diet quality by ethnicity/race among United States adults have been summarized in detail by Tao et al. [61]. Yet, studies investigating DAL among racial and ethnic groups are very scarce and only available for very specific populations (e.g. patients with renal disease) [19]. A comparative discussion with other studies is thus difficult, yet DAL is worthy of further investigations for its contribution to chronic disease outcomes across race/ethnic groups.

Essentially in line with the findings by Crews et al. [19], our results suggest higher DAL scores in Non-Hispanic Blacks than Non-Hispanic Whites. Yet, a numerical DAL score comparison between both studies may not expedient, because, unlike us, Crews et al. investigated patients with end-stage renal disease [19]. Of note, Crews et al. suggested that among US adults with chronic kidney disease, the association of DAL with progression to end-stage renal disease is stronger among Non-Hispanic Blacks than Non-Hispanic Whites.

Notably, the cross-sectional nature of our study is an intrinsic limitation and does not allow for such causal attributions. Our study did not include disease-specific endpoints but focused on descriptive recordings of DAL scores in ethnic/racial minorities.

Future prospective studies are thus warranted to gain a better understanding of DAL in ethnic/racial minorities. Such studies should preferably include food group analyses and investigate barriers to and opportunities for DAL-reduction strategies in the respective groups.

The present analysis draws upon a number of strengths. It is based on a nationally representative and large dataset from the National Health and Nutrition Examination Survey. The large sample size and the consideration of relevant confounders (e.g. special diets) are an additional asset. The topic is innovative and has not been analyzed before. Meanwhile, this analysis has several weaknesses worth mentioning, including the intrinsic limitations of a cross-sectional study, the inherent potential for various biases, and the lack of a food group analysis. Designed to investigate potential differences in DAL across ethnic/racial groups in the USA, this analysis does not allow for any causal interferences. Thus, it may not answer the question whether a high DAL actually contributes to ethnical/racial disparities in the USA. Although our statistical regression models took into account a number of potential confounders, we did not adjust for physical activity in order to avoid a substantial reduction in sample size. Finally, we excluded individuals on a special diet to reduce the likelihood of selection bias and to allow for a more homogenous comparison across ethnic groups. The various special diets captured in the NHANES yield different DAL scores [37]; by excluding individuals on special diets, we aimed to present a comparison of the ethnic groups on a standard Western diet. Yet, we acknowledge that in the more recent NHANES cycles, slightly more Non-Hispanic White adults were on a special diet than Non-Hispanic Black or Non-Hispanic Asian Adults [62].

## Conclusions

To the best of our knowledge, we present the first analysis to systematically investigate DAL scores across ethnic/racial minorities in the USA. Using data from the NHANES, we found substantial and significant DAL differences across the involved ethnic groups. Mexican Americans and Non-Hispanic Blacks had the highest DAL scores, with notable differences to Non-Hispanic Whites. Our analysis builds the foundation for future research in this area and raises many interesting research questions, including (but not limited to) as to whether these DAL differences could potentially play a role in population health inequity in the USA. Future prospective studies are urgently warranted to gain additional insights into this unexplored area of nutritional epidemiology.

### Supplementary Information

Below is the link to the electronic supplementary material.

Supplementary file1 (DOCX 25 KB)

## Data Availability

Data is publicly available online (https://wwwn.cdc.gov/nchs/nhanes/Default.aspx). The datasets used and analyzed during the current study are available from the corresponding author on reasonable request.

## Declarations

### Open Access

Open access publishing enabled by Project DEAL.

## Abbreviations

*NEAP*: Net endogenous acid production
*NCHS*: National Center for Health Statistics
*NHANES*: National Health and Nutrition Examination Surveys
*PRAL*: Potential renal acid load
