# Supplementary material for: Health Inequities in the USA: a Role for Dietary Acid Load? Results from the National Health and Nutrition Examination Surveys
Source: J Racial Ethn Health Disparities. 2022 Nov 23;10(6):2851–60. doi: 10.1007/s40615-022-01462-9 (PMC10645648; doi:10.1007/s40615-022-01462-9)
Supplement: Supplementary file 1 — Supplementary file1 (DOCX 25 KB) [file 40615_2022_1462_MOESM1_ESM.docx]

# Supplementary Tables

## Supplementary table 1

Supplementary table 1 title: Multivariate linear regression model examining potential associations between race/ethnicity and NEAP_R_

| Independent variables | β | linearized SE | p |
| --- | --- | --- | --- |
| Sex  Female | -14.37 | 0.46 | <0.001 |
| Age  30 – 39 years  40 – 49 years  50 – 59 years  60 – 69 years  70 years or older | 0.19  -1.57  -6.52  -9.03  -13.72 | 0.68  0.75  0.90  0.85  0.79 | 0.826  0.039  <0.001  <0.001  <0.001 |
| Ethnicity  Mexican American  Other Hispanic  Non-Hispanic Black  Other Race ^a^ | 2.64  -0.86  4.16  -5.46 | 0.80  0.86  0.65  0.71 | 0.001  0.318  <0.001  <0.001 |

Supplementary Table 1 legend: a = includes Multi-Racial. A significant regression equation was found: F(10,70) = 143.91, with a p-value < 0.001, and with an R2 values of 0.139. Reference categories were as follows: male sex, age 20 – 29 years, Non-Hispanic White.
